# Sex selection and non‐invasive prenatal testing: A review of current practices, evidence, and ethical issues

**DOI:** 10.1002/pd.5555

**Published:** 2019-10-10

**Authors:** Hilary Bowman‐Smart, Julian Savulescu, Christopher Gyngell, Cara Mand, Martin B. Delatycki

**Affiliations:** ^1^ Bruce Lefroy Centre Murdoch Children's Research Institute Melbourne Victoria Australia; ^2^ Department of Paediatrics University of Melbourne Melbourne Victoria Australia; ^3^ Uehiro Centre for Practical Ethics University of Oxford Oxford UK; ^4^ Victorian Clinical Genetics Services Murdoch Children's Research Institute Melbourne Victoria Australia

## Abstract

Non‐invasive prenatal testing (NIPT) can determine the sex of the fetus very accurately and very early in gestation. There are concerns that the ease, timing, and accuracy of NIPT sex determination will facilitate sex‐selective termination of pregnancy (TOP). Here, we review current practices, the evidence for a link between NIPT and sex‐selective TOP, and associated ethical issues. Sex‐selective TOP, usually motivated by son preference, has had serious demographic consequences in countries such as India and China. Currently, ultrasound is the primary method by which parents determine the sex of the fetus. The diffusion of ultrasound technology has had a direct impact on the rates of sex‐selective TOP. Although NIPT is currently more costly, it is feasible that increased uptake of this technology could have a similar effect. Partly because NIPT is a relatively recent development in prenatal screening, there is little data on the impact of NIPT on sex selection practices. Evidence that NIPT is playing a role in sex‐selective TOP remains largely anecdotal. Further research is required to assess and quantify TOP resulting from NIPT sex determination. The use of these technologies for sex selection raises a number of ethical issues, in addition to practical demographic consequences.

What's already known about this topic?

- Sex‐selective termination of pregnancy is a widespread practice in many areas of the world.
- It is commonly motivated by a son preference and possibly also for the purpose of “family balancing.”
- Non‐invasive prenatal testing can determine the sex very early in the pregnancy, and there are concerns that this will further facilitate sex‐selective termination of pregnancy.

What does this study add?

- Evidence that NIPT is facilitating sex selection remains anecdotal, and further research is required to quantify this.

## 1 INTRODUCTION

The introduction of non‐invasive prenatal testing (NIPT) has the potential to make the practice of sex‐selective termination of pregnancy (TOP) an even more pressing issue. NIPT can determine the sex of the fetus very accurately and very early in the pregnancy. It is increasingly accurate from 7 weeks' gestation.^1^ This is earlier than when other techniques that can determine sex, such as ultrasound or chorionic villus sampling (CVS), are generally performed.^2^, ^3^

There can be medical reasons to determine the sex of the fetus. For example, the mother may be a carrier of an X‐linked disorder such as Duchenne muscular dystrophy and may request further testing if the fetus is determined to be male, as if the fetus is male, there is a 50% chance he will be affected by the condition.^4^ However, it is also common for parents to want to find out the sex of the fetus for non‐medical (social) reasons.^5^ These reasons include planning and preparation, the desire for a closer emotional attachment to the fetus,^5^ or bestowing a sense of personhood and social identity upon the fetus that is perceived to be lesser when using gender‐neutral pronouns such as “it.”^6^

Sex determination can also be done for the purpose of sex‐selective TOP. Sex selection can be motivated by a cultural preference for one sex. There is evidence that sex selection in favour of males is occurring in several regions across the world, including India,^7^ China,^8^ and the Caucasus.^9^ Sex selection can also be motivated by for family balancing purposes.^10^ This is when a family has one or more children of one sex and wants the next child to be another sex in order to “balance” the family unit. Both rationales for sex selection raise complex ethical issues.

The aim of this paper is to review the current practice of sex‐selective TOP, the evidence for a relationship between NIPT and sex‐selective TOP, and the ethical issues associated with using NIPT to facilitate sex‐selective TOP. This review may be useful for clinicians and health professionals who are involved in the provision of NIPT sex determination, as well as informing policy makers who are considering the issue of prenatal sex determination.

## 2 NIPT AND SEX DETERMINATION

Non‐invasive prenatal testing (NIPT) was developed to screen for chromosomal conditions such as the trisomy disorders and, in some cases, microdeletions.^11^ NIPT can also determine the sex of the fetus early in the pregnancy. NIPT became available in Hong Kong and the United States in 2011, with a rapid spread in availability worldwide.^12^ NIPT has many advantages over other methods of screening and/or sex determination. It is non‐invasive (requiring only a blood sample from the mother) and thus carries fewer risks than invasive diagnostic techniques such as amniocentesis and chorionic villus sampling. It is also more accurate and can be done earlier than the other established screening option, combined first trimester screening (CFTS), which does not determine the sex of the fetus.^13^

NIPT is very accurate for the determination of fetal sex. A 2017 systematic review of NIPT in singleton pregnancies found that the sensitivity and specificity of NIPT for fetal sex is 0.989 and 0.996, respectively.^14^ NIPT is not offered by many providers for twin pregnancies, but it is possible to determine fetal sex of twins through NIPT with a predictive model producing an accurate prediction in 97.8% of cases,^15^ with a high sensitivity and specificity for the presence of a Y chromosome.^16^ Fetal sex determination can be performed through amplification of Y‐chromosome markers such as *SRY* and/or *DYS14* 17 using qPCR. A 2011 systematic review found that fetal sex determination by NIPT before 7 weeks gestation is unreliable.^1^ However, a more recent approach using ddPCR may lead to accurate fetal sex determination earlier than seven weeks of gestation.^18^ NIPT can also be used to indicate the presence of sex chromosome aneuploidies (SCAs) and other disorders of sex development.^19^, ^20^ However, it is important to note that the accuracy of NIPT for SCAs varies and is less accurate than its use for the autosomal chromosome disorders.^21^ Discordance between sex phenotype and NIPT results can also occur due to translocations, deletions, other disorders of sex development, vanishing twin syndrome, or maternal chimerism.^22^, ^23^

## 3 AVAILABILITY OF NIPT FOR SEX DETERMINATION

NIPT is currently being adopted in health care systems across the world. In many countries, NIPT remains a user‐pays option and is not publicly funded. However, some countries have begun to provide public funding for NIPT. In Belgium, NIPT has been available for reimbursement as a primary screening test since July 2017.^24^ In the Netherlands, NIPT is being provided as a screen to all pregnant women regardless of risk as part of the TRIDENT‐2 implementation study.^25^, ^26^ Other countries provide funding for NIPT contingent on risk for fetal anomalies, such as Switzerland,^27^ Denmark,^28^, ^29^ and Canada (only certain provinces).^30^, ^31^ In the United Kingdom, NIPT is available in the National Health Service (NHS) across Wales as a secondary screen for high‐risk populations.^32^ However, many of these systems do not fund non‐medical sex determination, instead focusing on screening for autosomal disorders.^33^, ^34^

NIPT for non‐medical sex determination is easily accessible through the private sector in many countries,^35^ but in some countries such as India, prenatal sex determination is outlawed.^36^ A 2017 Nuffield Council on Bioethics report recommended that non‐medical NIPT sex determination not be offered through the UK NHS or private providers.^35^ In the United Kingdom, there have also been calls by members of the Labour Party for non‐medical NIPT sex determination to be banned.^37^ In response, the Nuffield Council reiterated their stance that NIPT for non‐medical sex determination should not be offered through the NHS.^38^ They also recommended that private providers be prevented from providing non‐medical sex determination.

## 4 CURRENT METHODS OF SEX‐SELECTIVE TERMINATION OF PREGNANCY

There are concerns that NIPT, due to its ease, accuracy, and availability early in gestation, may facilitate an increase in sex‐selective termination of pregnancy (TOP).^35^ Ultrasound identification of sex is the current most common means of determining the sex, but its level of accuracy only approaches that of NIPT later in the pregnancy (eg, beyond 14 wk).^3^ NIPT can determine sex more accurately than ultrasound at earlier stages of gestation.^39^

An ultrasound is recommended at 18 to 22 weeks of gestation,^40^ and thus, this is the time in pregnancy where parent(s) will be most likely to determine the sex of their fetus, if they wish. However, pregnant women are increasingly requesting sex determination at a first‐trimester ultrasound (eg, 12 weeks' gestation), despite the lower accuracy of sex determination at this gestation.^3^ One 2016 study found that from the gestational age of 12 weeks, fetal sex determination was both feasible and accurate in 79% of cases; there is increased sensitivity in the determination of female sex compared with male sex.^41^ With increased gestational age (eg, beyond 14 wk), fetal sex determination becomes significantly more accurate than at 12 weeks' gestation.^3^

Invasive diagnostic tests such as CVS can also be used to determine fetal sex very accurately.^42^ However, generally, CVS is not performed before 10 weeks' gestation, due to an increased risk for limb malformation.^43^ The invasive nature of CVS also makes it a less desirable option than NIPT. It carries a risk, albeit small, of miscarriage.^44^

If sex‐selective TOP is the rationale behind sex determination, there is an incentive to determine the sex as early as possible, as TOP becomes more complex and expensive with increased gestational age.^45^ TOP at the time NIPT identifies fetal sex can be done by a surgical procedure, whereas by the time it can be confidently identified by ultrasound, TOP may require induction and delivery.^45^ In the early stages of gestation, NIPT is more accurate than ultrasound^39^ and also safer than invasive techniques such as CVS.^42^ TOP at earlier stages of gestation is also generally seen as more ethically acceptable due to the increasing moral status of the fetus as it grows and also may be associated with decreased psychological distress for the pregnant woman.^35^ Therefore, given that NIPT can allow for early, safe, and accurate sex determination, it is a possibility that this would make it easier to facilitate sex‐selective TOP.

## 5 SEX‐SELECTION: CURRENT PRACTICE AND MOTIVATIONS

Sex‐selective TOP is often driven by a strong son preference, resulting in the termination of female fetuses; this is particularly common in countries such as China or India, among others.^7^, ^8^ Sex‐selective TOP is one of the likely causes of male‐biased sex ratios in countries in Asia, Africa, and Eastern Europe.^46^ There are also significantly male‐biased sex ratios in the Caucasus region.^9^ The natural sex ratio is approximately 105 males for every 100 females, and major deviations from this are generally interpreted as evidence for prenatal or pre‐conception sex selection.^47^ Sex‐selective TOP is often biased against females and thus is a gendered issue.

In cultures without an overall strong son preference, such as the United States,^48^ sex‐selection (whether through termination or preimplantation genetic diagnosis) is commonly justified on the grounds of “family balancing,” where the parent(s) desire a mix of sexes in their children. Family balancing may therefore not involve an overall tendency towards one sex or the other—however, there are still ethical concerns.^10^ Regulation around sex‐selection for non‐medical reasons varies based on jurisdiction. The use of technologies such as sperm‐sorting^49^ and pre‐implantation genetic diagnosis^50^ is an established practice in regions where pre‐conception non‐medical sex‐selection is permitted. There is less evidence for the use of TOP as a method of sex‐selection for family balancing.

There are a number of reasons why a preference for male children exists in some regions. It is particularly prominent where patrilineal kinship systems incentivise the production of sons, and the family name is carried down through the male line.^51^ Another reason is economic. In India, the practice of a dowry for girls when they get married creates a significant cost for families.^52^ In China, sons are perceived to be a source of financial security.^53^ There can also be religious motivations for desiring a son.^54^, ^55^ In rural areas, sons can also be important as a source of agricultural labour.^56^ This preference has also been exacerbated by a decline in fertility in these regions—the fewer children a couple has, the more important it is that they do not solely have daughters.^57^

Sex‐selective TOP has had a number of negative demographic effects in countries such as China and India. For example, the large number of “missing women” in China has resulted in a large number of young men who are unable to find partners. This has resulted in decreased quality of life, impacted their social status, and increased competition among those who would like to find a female partner.^58^ This has also resulted in an increase in sex trafficking and bride trafficking from neighbouring regions and countries such as Vietnam.^59^

India and China have outlawed prenatal sex determination in an effort to prevent sex‐selective TOP. In India, this was done through the 1994 Pre‐Conception and Pre‐Natal Diagnostic Techniques (Prohibition Of Sex Selection) Act (PCPNDT Act).^60^ However, in India, this has not necessarily affected the widespread practice of sex‐selective TOP as abortion is legal, and these laws are often poorly enforced.^61^ It is important to note that female infanticide has a long history in India, but research suggests that in the modern era, the elimination of females has now largely shifted from infanticide to sex‐selective TOP.^62^ In China, this son preference is exacerbated by the historical One‐Child Policy, where in many regions of China, parents were until recently limited to one or possibly two children.^63^ However, it is a possibility that underreporting of female births due to this policy has also contributed to a skewed sex ratio at birth in the official statistics.^64^ As with India, prenatal sex determination is banned in China but remains a widespread practice, with enforcement proving to be difficult for the government.^65^ If a parent's firstborn child is a girl, they are more likely to undergo an ultrasound in subsequent pregnancies, which possibly indicates that there is widespread use of ultrasound for sex determination and selective TOP of female fetuses even though this practice is banned.^66^

## 6 CURRENT EVIDENCE FOR SEX SELECTION

**Figure 1:**
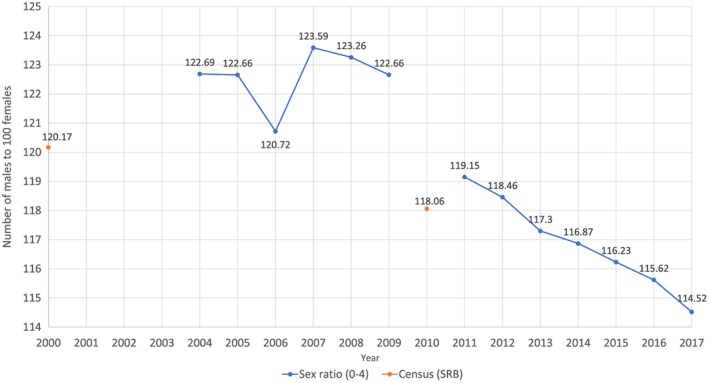
Data from National Bureau of Statistics of China (Annual Survey, 2000 & 2010 censuses)^68^ [Colour figure can be viewed at http://wileyonlinelibrary.com]

Access to ultrasound in regional areas in China was associated with an increase in the sex ratio at birth.^67^ Approximately 40% to 50% of the increase in sex ratio at birth from the 1980s onwards can be explained by access to sex determination through ultrasound.^67^ Although data for sex ratio at birth (SRB) are not available in China for non‐census years, there is an annual sample survey that provides data for the 0 to 4 age group (see Figure 1). For the census data, which does contain information about SRB, the SRB continued to slowly increase until the late 2000s.^69^ This is in accordance with the annual sample survey. However, while it is unclear how accurate the national statistics are,^70^ the data from the annual sample survey suggests that the SRB is now on a decline, although the most recent data reports that the SRB is still well above the natural ratio (at 114.5). This is in contrast to countries such as South Korea, which showed an increase in SRB in the early 1990s, but has since then exhibited a significant decline back to a natural ratio.^69^

**Figure 2:**
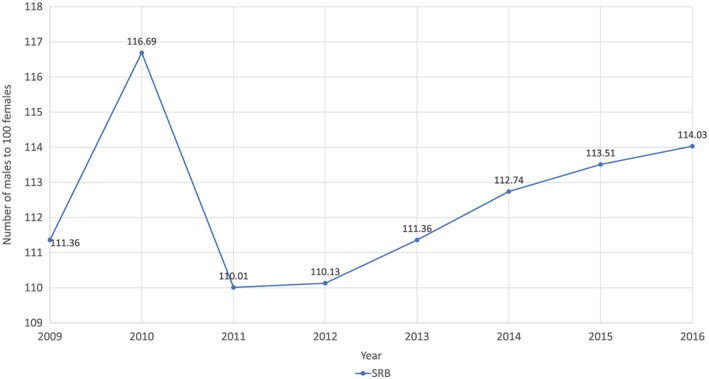
Sex ratio at birth from the Civil Registration System (India).^72^ N.B. data converted from females per 100 males (format of official figures) to males per 100 females [Colour figure can be viewed at http://wileyonlinelibrary.com]

In India, data from the Sample Registration System indicate that the SRB was declining up until 2011‐2013. However, the SRB for 2012‐2014 and 2013‐2015 showed a marked increase in the SRB.^71^ The Civil Registration System, which has recorded above 80% of births since 2011, shows an even more marked increase in the SRB, from 110 (2011) to 114 (2016) (rounded figures) (see Figure 2).^73^

Sex‐selective TOP may also be occurring in countries with population‐level balanced SRB. A 2015 assessment of the issue of sex‐selective TOP in the United Kingdom, by the Department of Health, found that there was no evidence of sex‐selective TOP occurring.^74^ However, this is in contrast to previous research from 2007 that found an increased SRB among Indian‐born mothers in the United Kingdom, especially with multiparous women.^75^ Research examining data from 2007‐2011 did not, on the other hand, find any skewed SRB among Indian‐born women, although there was an effect in mothers born in Southeast Asia and the Middle East^76^; it is important to note that this latter study did not break the ratios down by parity.

Similar male‐biased SRB have been observed in immigrant populations of other countries, particularly the Indian‐ and/or Chinese‐born populations of Spain,^77^, ^78^ Canada,^79^, ^80^, ^81^, ^82^, ^83^, ^84^ (including second‐generation mothers^85^), Norway^86^ (although some research contradicts this^87^), Sweden,^88^ Greece,^89^, ^90^ Italy,^91^ Australia,^92^ and the United States.^48^, ^93^, ^94^, ^95^, ^96^, ^97^, ^98^, ^99^ These trends are most evident with higher birth orders; women in these demographics with two or more girls are generally much more likely to have a boy in following pregnancies. This suggests that son preference does not influence termination of earlier pregnancies as much as it does when previous pregnancies have been completed and have resulted in girls.

## 7 NIPT SEX DETERMINATION AND SEX SELECTION

Much of this data precedes the advent of NIPT. It is unclear if changes in SRB indicating sex‐selection will be influenced by the ease of access of NIPT. More research for the period from 2012 onwards is necessary to be able to examine if there is a possible impact of NIPT on SRB. In Australia, an analysis of all live births in the state of Victoria showed an SRB of 1.25 for multiparous women born in China, in the period of 2011‐2015. For multiparous women born in India, the SRB was 1.22. This was higher in comparison with previous years and also in comparison with the population SRB of 1.05 for multiparous women in 2011‐2015.^92^This increase in SRB from 2011 onwards approximately correlates with the introduction of NIPT and so may indicate a role that NIPT is playing in sex‐selective TOP. There are anecdotal reports that NIPT is being used to facilitate sex‐selective TOP in the United Kingdom,^37^ with a BBC report in September 2018 highlighting this possibility.^100^ One participant in a 2018 survey of genetic counsellors in the United States reported that “We have had couples terminate for gender. NIPT gets them results early enough they can act without people knowing they are pregnant … .”^101^

As noted previously, the SRB is increasing in India. However, it is important to note that while NIPT in India is available, prenatal sex determination is illegal. This includes prenatal sex determination through NIPT for all Indian‐based laboratories. NIPT first became used in India via home tests sent to laboratories in the United States,^61^ which would bypass the legislation against prenatal sex determination. This limited the use of the test to those with the means to access such testing. Therefore, it is not likely that NIPT has had a large impact on the sex ratio in this time. However, as explored earlier, the diffusion of a technology that could easily determine sex prenatally (ultrasound) had a serious impact on the level of sex‐selection. It is feasible that the introduction of a newer, more accurate technology (NIPT) could follow a similar path in spite of the ban on prenatal sex determination.

There is some empirical research into the views of women (including previous users of NIPT) towards the use of NIPT for non‐medical sex determination and selection. One 2014 study from Ohio, in the United States, found that 49% of respondents (pregnant women) would use NIPT for sex determination, as compared with CVS (19%).^102^ A 2016 Swedish study found that 50% of respondents (pregnant women) would like to use NIPT to find out the fetal sex.^103^ A more recent 2019 study from Victoria, Australia, which surveyed women who had previously undergone NIPT, found that 61% indicated an interest in using NIPT for sex determination, although a higher percentage (86%) indicated support for the general availability of NIPT for such a use. When asked about the use of that information for sex‐selective TOP, 99% of respondents indicated that they would “definitely not” consider it.^104^ In comparison with these relatively supportive values for NIPT sex determination, a 2015 study from the Netherlands found that only 19% of pregnant women surveyed strongly disagreed with the assertion that NIPT “should only be possible for disorders, so not for gender” (54% neither disagreed or agreed).^105^ A study of online discussions about NIPT found that some women reported using NIPT specifically for sex determination, although they did not disclose this to their health care provider as their primary motivation.^106^

Health care providers have highlighted that their patients often wish to know the sex of the fetus as early as possible, finding NIPT useful for this purpose.^107^ A 2019 survey of European health care providers found varying levels of interest (on the part of the providers) in the use of NIPT to determine sex chromosomes, from 51% in France to 78% in Spain. It is important to note that this study did not specify non‐medical sex determination.^108^ A 2018 study of 103 genetic counsellors in the United States found that 60% offered NIPT for determination of sex chromosomes to all patients, and there were no participants who did not offer it at all. Seventy‐five percent of participants thought it should be offered universally, although the percentage of those concerned about the use of NIPT for sex determination had increased significantly since a similar survey in 2015 from 1% to 26.5%.^101^ In another study from the United States, a 2016 survey of maternal‐fetal medicine fellows, 54.4% of respondents believed that it was somewhat or very likely that NIPT would lead to an increase in sex‐selective TOP.^109^ Research from 2016 into the views of Pakistani obstetricians found that 55% would not offer NIPT for sex determination. However, 31% would offer this, despite recognising a strong son preference, with participants suggesting the illegal status of abortion for social reasons in Pakistan as sufficient protection against sex‐selective TOP.^110^ A 2017 survey of obstetric professionals in New Zealand found that 37% had been asked about using NIPT for sex determination, and 33% supported the public funding of NIPT sex determination.^111^

## 8 ETHICAL ISSUES

The primary ethical objections to the use of NIPT for sex determination stem from its possible use as a tool for sex‐selection. Many of these objections apply to sex determination and sex‐selective TOP in general. However, as previously stated, NIPT intensifies these concerns due to its accuracy, ease of use, safety, and availability early in gestation. The early accuracy of NIPT removes much of the uncertainty that other methods such as ultrasound still have and thus can result in more informed decisions and removes the risk of inaccurate results that can temper decision‐making around sex‐selective TOP. NIPT also allows for sex‐selective TOP in the first trimester, which is easier in practical terms, as well as allowing the pregnant woman to delay maternal‐fetal bonding. These factors may make sex‐selective TOP significantly easier to pursue if NIPT is used. Therefore, it is important to explore these ethical issues in the context of NIPT adoption.

Widespread sex‐selection could lead to harms on the societal level, which are already experienced by countries with significantly skewed sex ratios due to a strong cultural son preference. However, beyond the practical consequences of sex‐selection on a wider scale, there are also ethical concerns about sex‐selection as a choice that individuals make. This includes the use of sex‐selection as a tool for “family balancing,” when the choice is not necessarily driven by a strong son preference.

### 8.1 Autonomy

A common argument for sex selection is from respect for reproductive autonomy and liberty. Allowing for sex selection (in this case, through NIPT and sex‐selective TOP) would satisfy the preferences of those parents who desire a child of one particular sex and would not otherwise reproduce if they could not realise those preferences.^112^ From this perspective, sex selection is a legitimate exercise of reproductive choice and parents should not be limited from satisfying their preferences.^113^ NIPT can allow for increased autonomy in reproductive decision‐making due to its high accuracy, which allows for more informed choices. Sex‐selection through NIPT does not carry any risk of physical harm to the fetus if it is carried to term, and so we do not need to be concerned with previous fears about harms to future persons due to the nature of sex selection (eg, sperm sorting). Although there may be harms to the child that accrue from parental expectations, there is not necessarily evidence that these harms outweigh the benefit gained from parents realising their preferences (as parents always have expectations^113^). However, an objection to this is it dislocates the individual choice of the parents from the social context in which they make their choice, and that from the perspective of relational autonomy theorists, these reproductive choices cannot be understood solely on an individual level.^46^ This means that an individual's choice is irrevocably intertwined with and shaped by the social context (such as gender inequity) in which they make their choice—a choice that can further reinforce these inequities. Another argument is that sex selection in fact *undermines* reproductive autonomy because it is predicated on a false belief (ie, that the child will definitely exhibit the expected gendered behaviours) and prevents parents from fulfilling their desired goals, such as engaging in masculine‐coded pastimes if they have a daughter, on the grounds of this mistaken belief.^114^

### 8.2 Sexism

Powledge has described prenatal and pre‐conception sex selection as “the original sexist sin.”^115^ Sex selection is sexist because it makes a judgement of value based solely on the attribute of sex alone. The choice to select sex can also, it is argued, perpetuate sexist discrimination.^116^ Although some argue that allowing family balancing in Western countries would not result in biased sex ratios, due to societal gender equity, Hendl objects that sex selection based on predetermined sex roles is already an indicator of a society that has not achieved gender equity.^46^ From this perspective, the sexism inherent in sex selection is not necessarily limited to male supremacy (as expressed by son preference), but rather rooted in notions of gender essentialism.^117^ The harmful sexist views, therefore, can be sex stereotyping and not just sex supremacy.^118^ Some empirical research suggests that desires for family balancing are primarily motivated by parents envisioning a particular kind of relationship with a child of a particular sex.^117^ Some theorists have argued that the belief that a parent will have a particular relationship based on the sex of the child is a sexist belief that in itself could constitute a form of harm to the child.^119^ However, other theorists argue that this is not necessarily indicative of sexism, but rather a means of parents seeking a diversity in their parent‐child relationships, and that this is not harmful to anyone.^120^

### 8.3 Commodification

Another objection to sex selection is that it is a form of “designing” or “choosing” children and thus commodifies them and subjects them to certain expectations, rather than viewing them as individuals.^121^ Similarly, another objection to family balancing is that it medicalises the family unit, socially constructing families with multiple children of the same sex as “imbalanced,” and thus wrong or pathologised in some way.^122^

### 8.4 Objections to sex determination itself

There are some feminist critiques of prenatal sex determination in general. One argument from Browne is that providing prenatal sex determination through NIPT is wrong because it encourages the conflation of sex and gender and thus provides a kind of “misinformation” and that the societal benefits that accrue from undermining gender essentialist beliefs outweigh the parent's right to know the sex of their fetus (especially at the early stage of gestation that NIPT allows for).^123^ Providing sex determination early in gestation, as NIPT allows for, also alters consumption patterns and leads the creation of a gendered environment through objects (such as toys or clothing) before the baby is even born. This phenomenon is in fact relatively recent and has been facilitated by the availability of prenatal sex determination that is accessible earlier and earlier.^6^ This prenatal gendered consumption has the possibility of unnecessarily socially reinforcing gender norms and stereotypes.

## 9 THE FUTURE OF NIPT AND SEX SELECTION

It is unclear as of yet what impact NIPT will have on trends for sex‐selective TOP. There is anecdotal evidence that NIPT is associated with sex‐selective TOP. However, more rigorous evidence is required to determine whether this is indeed occurring and how often. In addition, it is unclear whether NIPT is associated with an *increase* in sex‐selective TOP, or if it would merely be a different means. It is unknown whether NIPT facilitates sex‐selective TOP more or less than other methods, such as ultrasound and invasive prenatal testing. In addition, as NIPT is less widely accessible than other methods such as ultrasound, it is important to establish the extent to which NIPT contributes to sex‐selective TOP in the context of its availability.

Early sex determination through NIPT raises a number of ethical issues. Many of the ethical issues apply to sex‐selective TOP and sex determination in general. However, NIPT intensifies these issues do its ease, safety, and availability early in gestation. Nevertheless, the lack of good evidence for NIPT facilitating sex‐selective TOP should inform the ethical debates, particularly until more conclusive evidence relating to this can be produced.

Further research is required into the SRB of children born following NIPT to more accurately ascertain whether NIPT use is associated with a bias towards one particular sex. Additional research could also examine the motivations of those using NIPT for sex determination and determine whether sex‐selection forms any part of these. While sex‐selective TOP and skewed SRBs remain a serious issue globally, it is not immediately obvious what role NIPT will play in this problem in the future.

## CONFLICTS OF INTEREST

MBD is the Clinical Director of the Victorian Clinical Genetics Services, a not‐for‐profit organisation that provides the *percept* NIPT.

## FUNDING SOURCES

Research conducted at the Murdoch Children's Research Institute is supported by the Victorian Government's Operational Infrastructure Support Program. This work was supported by the Wellcome Trust (203132). This research was supported by an Australian Government Research Training Program (RTP) Scholarship (HBS).

10

## Data Availability

Research data are not shared.
